# Repression of Marriage of Criminals

**Published:** 1901-11

**Authors:** 


### EDITORIAL NOTES AND COMMENTS.

The Business office of THE JOURNAL-RECORD is No. 64 Marietta Street.

The Editorial office is 318-319-320 The Prudential.

Address all Business communications to Dr. M. B. Hutchins, Mgr.

Make remittances payable to THE ATLANTA JOURNAL-RECORD OF MEDICINE.

On matters pertaining to the Editorial and Original Communications address Dr. Bernard Wolff, Atlanta.

Reprints of original articles will be furnished at cost price. Requests for the same should always be made *on the manuscript*.

We will present, post-paid, on request, to each contributor of an original article, twenty (20) marked copies of THE JOURNAL-RECORD containing such article.

### REPRESSION OF MARRIAGE OF CRIMINALS.

ELSEWHERE in this issue of THE JOURNAL-RECORD will be found the recommendations of the Sociological Committee of the Tri-State Medical Society of Tennessee, Alabama and Georgia. The question concerned is one of great importance, and the proper solution of it is of high interest to mankind. The problem especially elicits the attention of the medical profession, for in its keeping falls so much of the responsibility of the moral and physical uplifting of the human species. A man's moral nature must be very dead indeed if he fails to appreciate the justice and wisdom of the recommendations of this committee, and it is the earnest wish of every right-thinking man and woman that much good may proceed from the movement. Yet at the threshold, the thought arises, is the plan suggested feasible? Man is swayed more by the sentiments than by reason, by the heart than by the head, and it is therefore doubtful whether laws and legislative enactments which are unable to suppress the unsentimental crimes of theft, murder, arson and the like can effect much in the regulation of the marriage of the unfit, where love or whatever the mawkish sentiment be termed, is first in evidence and the influencing motive. When legal restrictions are placed upon a certain thing it immediately becomes more attractive and desirable than it ever was before. Among the criminal classes concubinage is much more the rule than marriage, and even when the latter exists, it is merely an incident and the bonds are too frail and loose to offer resistance to separation. Under such conditions laws prohibitive of marriage would be merely empty forms. Cohabitation without marriage is already illegal, but do men and women any the less cease to cohabit? Further, criminality is not limited to the lower classes, for in the higher walks of life exists a more insidious because a more refined type of viciousness, and one that is as surely transmitted to offspring as are the cruder forms of moral delinquency among the submerged. The absconding Sunday-school superintendent, the defaulting bank official, the dishonest merchant, are they molested by law in relation to whom they shall marry or whether they shall be allowed to marry? Not a bit of it. If detected they live out or work out their crime, and then if any woman is willing to take them, there is always a license and a preacher to be had for the price. It is a poor law that doesn't work in all directions. The purification of criminality is, as Ingersoll said of politics, an iridescent dream. Another deluge or a repetition of the fate that overtook the cities of the plain are the proper remedies, or castration and ovariotomy as a substitute for fire and flood.

---

IN the death of Dr. G. G. Roy on October 18th, Atlanta has lost one of her best known physicians and most useful citizens. Dr. Roy had been in bad health for some years, but was able to attend to his practice to within a short time before his death. He was sixty-five years of age and was born in Essex county, Virginia. He came to Georgia during the war and was assigned to duty at the prison at Andersonville, where he rendered valuable service. He located in Atlanta and for years enjoyed a large and lucrative practice. He was professor of materia medica in the Southern Medical College up to the time of its consolidation with the Atlanta Medical College. He was several times a city councilman. Dr. Roy was a true friend, an excellent physician and an upright, pious and honorable man. He leaves a widow and one son, Dr. Dunbar Roy, to mourn his loss.

---

THE first annual meeting of the fellows and members of the Georgia Pasteur Institute was held in Atlanta, October 4th. The President, Dr. Henry R. Slack, read his address, which was highly satisfactory to the fellows. Twenty-two cases had been treated. Fifteen proved by inoculation to have been bitten by rabid dogs, and death of other animals. All improved under treatment and are now well. The institute is now well established and free of debt. The following board of governors and officers were elected: President, Dr. Henry R. Slack, LaGrange; Vice-President, Dr. J. H. McDuffie, Columbus; Treasurer, Dr. C. D. Hurt; Secretary and Pathologist, Dr. Claude A. Smith; Physician, Dr. James N. Brawner, Atlanta; Dr. E. P. Ham, Gainesville; Dr. M. M. Holland, Statesboro; Mr. Benj. W. Hunt, Eatonton, and Mr. George C. Walters, Atlanta.

---

THE trustees of the State Sanitorium at Milledgeville met for their annual meeting, October 17. The resignations of Drs. W. A. O'Daniel and Job C. Patterson were accepted. Dr. T. O. Powell was elected superintendent; Dr. James M. Whitaker, first assistant physician, and Drs. Ladrick, M. Jones, John W. Mobley, Middleton L. Perry, N. P. Walker, Augusta, and E. M. Green, Danville, Ky., were elected assistant physicians.

